# Remarkable improvement of primary orthostatic tremor using perampanel

**DOI:** 10.1186/s42466-020-0050-0

**Published:** 2020-01-29

**Authors:** Marcus Grobe-Einsler, Oliver Kaut

**Affiliations:** 1 Department of Neurology, University Clinic Bonn, Venusberg-Campus 1, 53105 Bonn, Germany; 2 German Center for Neurodegenerative Diseases (DZNE), Bonn, Germany

**Keywords:** Orthostatic tremor, Tremor, Perampanel

## Abstract

Management of primary orthostatic tremor (POT) remains challenging, and medication is often ineffective.

We report the case of a 53-year-old female with orthostatic tremor for 6 years who was refractory to gabapentin, clonazepam, primidone and propranolol. After treatment with 4 mg/day perampanel, she reported almost complete resolution of tremor. The diagnosis of POT was confirmed by tremor analysis using surface electromyography.

Our report shows the potential use of the novel AMPA (α-amino-3-hydroxy-5-methyl-4-isoxazolepropionic acid) receptor antagonist perampanel for the treatment of POT. To date, only two similar patients, one refractory to treatment and the other previously treated with clonazepam only, have been reported. We would like to note that our patient was refractory to all previous therapy and responded to a low dose of perampanel without side effects. The striking clinical improvement suggests a putative role of glutamate in the pathophysiology of orthostatic tremor.

## Introduction

Primary orthostatic tremor is a rare neurologic movement disorder characterized by unsteadiness in gait and (more pronouncedly) in stance due to a pathognomonic 13–18 Hz tremor that can be detected by surface EMG of both legs while standing. Involvement of the upper extremities is less frequent but has also been described. The pathophysiology of POT remains poorly understood, and limited treatment options are available. To date, multiple therapeutic approaches, of which gabapentin, benzodiazepines, primidone, propanolol, pregabalin and phenobarbital have been shown to be likely or possibly beneficial, have been tried [8]. The clinical response is frequently low, and the non-responder rate remains high [2]. We herein report a 53-year-old female with a clinical and electrophysiological diagnosis of medication-resistant orthostatic tremor who experienced significant improvement under therapy with perampanel.

## Case

The first symptoms occurred 6 years before diagnosis; they occurred at high frequency and included low amplitude tremor seconds after standing up that was less pronounced while walking and suspended during rest. POT was diagnosed based on surface EMG. Upper extremity functions were not impaired at the time of diagnosis. The only comorbidity was recurrent depressive episodes beginning 5 years before the onset of tremor. Treatment with gabapentin (2400 mg/day), clonazepam (4 mg/day), primidone (500 mg/day) and propanolol (unknown dose) showed an insufficient clinical response with disease progression. In parallel, depressive episodes worsened as the tremor progressed.

**Fig. 1:**
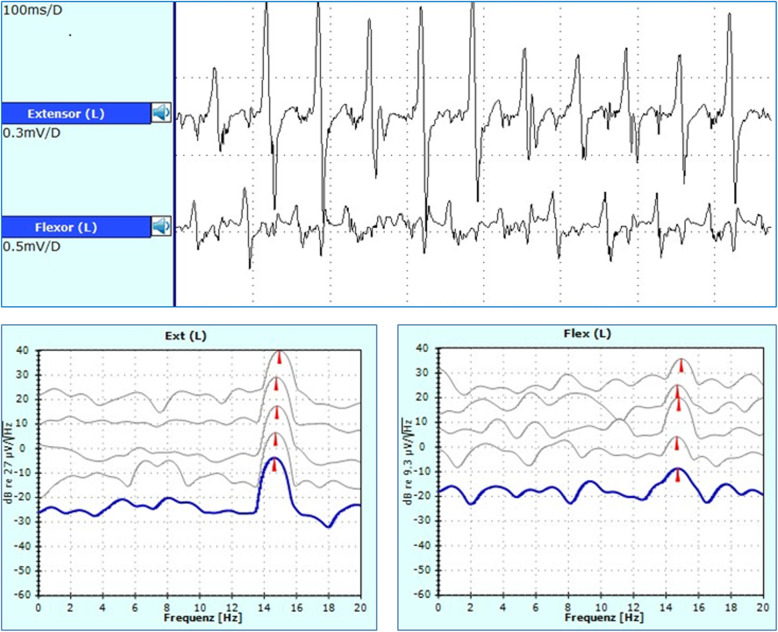
Upper lane: Surface electromyography (EMG) of the left quadriceps femoris (extensor) and biceps femoris (flexor) after treatment with 4 mg/day perampanel. Tremor (14–16 Hz) in standing position was arrested after sitting down. Compared to previous EMG (not shown), tremor showed delayed onset. Lower lane: The frequency spectrum demonstrated a peak between 14 and 16 Hz for both antagonists

In 2019, the patient frequently depended on support while walking and was incapable of free stance. Fine motor functions were impaired by tremor of the upper extremities while writing. No further neurological abnormalities were detected. Due to insufficient clinical response to standard therapy, a joint decision for off-label therapy with perampanel was obtained. Therapy was started after the cessation of premedication at a starting dose of 2 mg/day in the evening, and the dose was raised to 4 mg/day. Perampanel led to significant improvement of tremor with recovery of free stance and walking. The patient was able to walk freely for a couple of kilometres. The treatment effect was confirmed by persistent but delayed tremor after standing up, as demonstrated by electrophysiological testing (Fig. 1). Two months after the initiation of treatment, the patient reported a persistent treatment effect with only minor fatigue as a side effect.

## Discussion

The precise pathophysiology of orthostatic tremor and the mechanism of action of frequently used drugs remain unknown. The presence of a central oscillatory network and pathology in the cerebello-thalamo-cortical network have been discussed [3]. Many drugs that are frequently used for POT may act by reducing hyper-excitability. Gabapentin has been shown to be partially effective for POT [4, 5]. It is speculated to act via its anti-glutamatergic effects rather than its GABAergic effects. Glutamate represents the most prevalent neurotransmitter in the human brain and the most common excitatory neurotransmitter in the cerebellum. Its receptor subtypes are NMDA (N-methyl-D-aspartate) and AMPA (α-amino-3-hydroxy-5-methyl-4-isoxazolepropionic acid) receptors. These receptors are often present together in excitatory synapses and therefor interact in many ways. Glutamate has already been associated with clinically different forms of tremor, such as essential tremor (ET) [1] and tremor in Parkinson’s disease (PD), for which amantadine (a weak antagonist of NMDA receptors) is frequently prescribed. To date, the contribution of glutamate to the pathophysiology of orthostatic tremor is elusive.

Perampanel is a first-in-class selective, non-competitive AMPA receptor antagonist that is approved for epileptic seizures. Similar to amantadine, perampanel also blocks glutamate function. In a trial enrolling 12 patients with ET, perampanel (4 mg/d) led to significant improvement of tremor [1]. Perampanel was also shown to exert positive effects on orthostatic tremor in two case reports, one involving a patient with therapy-refractory POT [6] and one involving a patient who did not respond to treatment with clonazepam alone (20 mg/day) [7]. Electrophysiological testing showed persistent but reduced tremor in both patients. Both case reports did not report significant side effects of perampanel, but 4 of the 12 ET patients stopped medication due to intolerable unsteadiness, dizziness and nausea. Our patient suffered from drug-resistant POT, and perampanel led to a remarkable clinical improvement without clinically significant side effects. Only minor fatigue was reported during the follow-up.

To our knowledge, this is the third reported case of significant improvement of POT under treatment with perampanel and the second case involving medication-resistant POT. Adverse events such as the deterioration of psychiatric symptoms should be discussed with every patient treated with perampanel especially because of the frequent psychiatric comorbidities in patients with POT. The striking clinical improvement in all three reported patients suggests a central role of glutamate in the pathophysiology of POT and indicates the potential benefits of treating POT patients with perampanel. Randomized controlled trials are needed to evaluate the potential therapeutic effect.

## Data Availability

The datasets generated and/or analysed during the current study are not publicly available due to data protection regulations (clinical data) but extracts are available from the corresponding author on reasonable request.

## Abbreviations

AMPA: α-amino-3-hydroxy-5-methyl-4-isoxazolepropionic acid
EMG: Electromyography
ET: Essential tremor
NMDA: N-Methy-D-Aspartat
PD: Parkinson’s disease
POT: Primary orthostatic tremor
